# The P-SSP7 Cyanophage Has a Linear Genome with Direct Terminal Repeats

**DOI:** 10.1371/journal.pone.0036710

**Published:** 2012-05-11

**Authors:** Gazalah Sabehi, Debbie Lindell

**Affiliations:** Faculty of Biology, Technion – Israel Institute of Technology, Haifa, Israel; Tel Aviv University, Israel

## Abstract

P-SSP7 is a T7-like phage that infects the cyanobacterium *Prochlorococcus* MED4. MED4 is a member of the high-light-adapted *Prochlorococcus* ecotypes that are abundant in the surface oceans and contribute significantly to primary production. P-SSP7 has become a model system for the investigation of T7-like phages that infect *Prochlorococcus*. It was classified as T7-like based on genome content and organization. However, because its genome assembled as a circular molecule, it was thought to be circularly permuted and to lack the direct terminal repeats found in other T7-like phages. Here we sequenced the ends of the P-SSP7 genome and found that the genome map is linear and contains a 206 bp repeat at both genome ends. Furthermore, we found that a 728 bp region of the genome originally placed downstream of the last ORF is actually located upstream of the first ORF on the genome map. These findings suggest that P-SSP7 is likely to use the direct terminal repeats for genome replication and packaging in a similar manner to other T7-like phages. Moreover, these results highlight the importance of experimentally verifying the ends of phage genomes, and will facilitate the use of P-SSP7 as a model for the correct assembly and end determination of the many T7-like phages isolated from the marine environment that are currently being sequenced.

## Introduction

T7-like podoviruses that infect cyanobacteria are often isolated on high-light-adapted *Prochlorococcus* ecotypes [1], abundant unicellular cyanobacteria that contribute significantly to primary production in the oceans [2]. Furthermore, metagenomic analyses indicate that T7-like cyanophages are common in marine environments [3], [4]. The study of ecologically relevant model host-phage systems is of great importance if we are to gain an understanding of the impact phages have on the population dynamics, genome diversity and evolution of their cyanobacterial hosts.

P-SSP7 is such a model cyanophage that infects *Prochlorococcus* sp. strain MED4 [1]. The genome of this phage has been sequenced and determined to be T7-like with respect to its genome content and organization [5] as well as its transcriptional program during infection [6]. In addition to a common set of T7-like core genes this phage encodes *psbA,* a host-like photosynthesis gene often found in cyanophages [7], [8], [9], [10]. This gene is expressed during infection and is thought to be involved in the energy production needed for maximal phage replication [11]. It is also one of a few marine phages for which structural analyses of the phage particle have been carried out [12].

The genomes of T7-like phages typically contain direct terminal repeats that are used during genome replication [13], including at least one T7-like cyanophage that infects *Synechococcus* [14]. These repeats serve as regions of homology for recombination among near-complete genomes and lead to the formation of linear concatemers [13]. These concatemers are cleaved in a sequence-specific manner at the end of the repeat region at the right side of the genome during packaging [13]. While the mechanism for the completion of the left repeat region prior to packaging is still not fully understood [13], the result is that each phage particle contains a full-length genome with fixed ends and nonpermuted terminal redundancy in the form of direct repeats [13], [15].

The genome of P-SSP7 was reported to be 44.97 kb in size and to contain 54 open reading frames (ORFs) [5]. The genome assembled as a circular chromosome, therefore it was hypothesized that it is circularly permuted and lacks the direct terminal repeats found in other T7-like phages [5]. The lack of direct terminal repeats in P-SSP7 would imply that this phage employs a different mechanism of replication and packaging than other T7-like phages. However, as indicated when put forward, the confirmation of this hypothesis would require experimental verification through direct sequencing of the genome ends of P-SSP7 [5].

Here we experimentally determined the genome ends of the P-SSP7 genome and found that, similar to other T7-like phages, it has fixed ends with direct terminal repeats. This indicates that P-SSP7 is likely to undergo replication and packaging in a manner similar to other T7-like phages.

## Results

**Figure 1:**
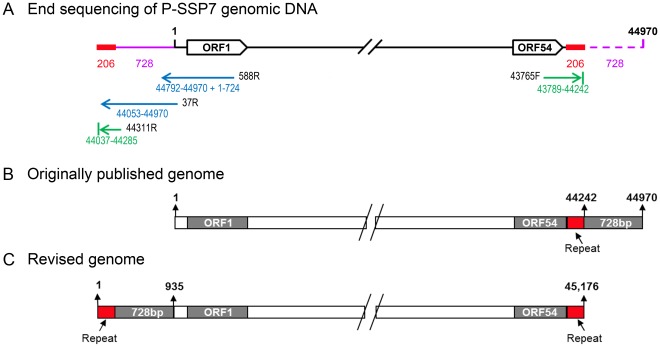
Schematic illustration of the arrangement of the P-SSP7 genome. (A) Sequencing of the ends of the P-SSP7 genome extracted directly from phage particles. Arrows, and numbers under the arrows, indicate the sequences acquired: Blue from the entire genome and green from end fragments produced by digestion of the genome with the BamHI and PmeI restriction enzymes. The positions of the primers used for sequencing are shown in black type at the beginning of the arrows. Genome numbering for the primers and sequences is that for the originally published sequence [5]. The purple line denotes the 728 bp region found to be upstream of ORF1 in this study, but positioned downstream of ORF54 in the originally published sequence. The repeat regions are shown in red at both ends of the genome. (B) Diagram showing the arrangement of the P-SSP7 genome as originally published (GenBank accession numbers: AY939843.1, [5] and GU071093 [16]. (C) Diagram of the revised genome arrangement based on the results from this study (updated GeneBank submission, accession number: AY939843.2).

In order to assess whether the P-SSP7 phage genome has discrete termini, we used high molecular weight DNA extracted from phage particles to sequence its ends. We used both undigested DNA as well as DNA that was digested with the BamHI and PmeI restriction enzymes. The sequence at the left end of the genome map was determined using a series of three primers, the first of which extends outwards from ORF1 (Fig. 1A). The right end of the genome map was determined using a single primer that extends outwards from ORF54. Sequence analysis revealed that the right-hand side of the genome ended 728 bp earlier than previously reported [5]. This 728 bp region was found to be located upstream of ORF1 at the left-hand side of the genome map (Fig. 1A). Furthermore, the left-hand terminus of the genome contained an additional 206 bp which were identical to the 206 bp region located at the right-hand terminus of the genome (Fig. 1A). The same results were obtained with end sequencing of the P-SSP7 genome cloned into a fosmid vector except for 7 bp that were missing from the right end of the cloned genome (data not shown). These results indicate that the genome extremities end at discrete positions and that a 206 bp repeat region is present at both termini.

**Figure 2:**
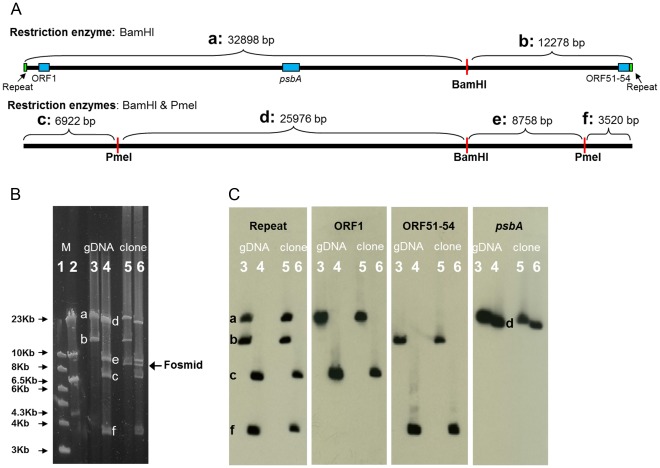
Digestion and Southern analyses of the P-SSP7 genome. (A) Schematic genome map showing the positions of the restriction enzyme cleavage sites (red) and the expected fragment sizes after digestion with BamHI alone (top) and both BamHI and PmeI (bottom) based on the revised genome arrangement shown in Fig. 1C. (B) Restriction digestion of the P-SSP7 genome extracted from phage particles (lanes 3 and 4) and the genome cloned into a fosmid (lanes 5 and 6), with BamHI alone (lanes 3 and 5) or with BamHI and PmeI (lanes 4 and 6), separated by pulse field gel electrophoresis. Note that the only difference for digestion of the cloned genome is the presence of an additional fragment corresponding to the size of the fosmid vector. Fragments corresponding to the expected sizes shown in (A) are marked with the appropriate letter designations (a to f). Fragment size markers (M): 1 kb DNA ladder (lane 1) and Lambda DNA cut with HindIII (lane 2), are shown. (C) Southern analyses of the restriction digested DNA in (B) using 4 probes (denoted above the lanes) show that the repeat region appears twice on the genome on the same fragments as the first and last ORFs. The positions of the gene probes on the genome are shown as light blue boxes and the repeat region probe as green boxes in the top panel of (A). Lane numbering and fragment designations are the same as in (B).

Digestion of the phage genome with BamHI or BamHI and PmeI resulted in discrete restriction fragments (Fig. 2B), indicating that the genome has fixed ends and is not circularly permuted. The sizes of the terminal fragments were greater than 6.5 kb (fragment c) and smaller than 4 kb (fragment f) (Fig. 2B). These sizes are consistent with the 728 bp region being located upstream of ORF1 rather than downstream of ORF54. The results were the same for the DNA extracted directly from phage particles and the genome cloned into the fosmid.

Southern analyses of phage genomic DNA extracted directly from phage particles as well as of the genome cloned into the fosmid provided final confirmation of the organization of the P-SSP7 genome. A probe for the repeat region confirmed that it is present at two places in the genome (Fig. 2C). The use of additional probes indicated that these repeat regions are present on the same two fragments that contain ORF1 (fragment c) and ORF51-ORF54 (fragment f) and separate from the large central part of the genome that contains the *psbA* gene (fragment d).

## Discussion

The results presented here indicate that the P-SSP7 genome is a linear molecule of dsDNA with fixed ends and direct terminal repeats of 206 bp, rather than being circularly permuted as was previously suggested [5]. The additional repeat of 206 bp means that the genome is 45,176 bp long rather than 44,970 bp. A 728 bp region of the genome, originally thought to lie downstream of ORF54, is actually located upstream of ORF1. It should be noted that the version of the P-SSP7 genome sequenced using 454 pyrosequencing technology and assembled with the Newbler assembly program (Genbank accession GU071093) [16], contains the same assembly error as the original P-SSP7 genome sequence for which 1–8 kb shotgun clone libraries were used in Sanger sequencing (Genbank accession AY939843) [5].

The order and arrangement of the open reading frames of this genome are the same as previously published [5] and are not affected by the reorganization of the terminal regions of the genome. However an additional 3 ORFs (ORF19A, ORF20A, ORF20B) were subsequently identified and the start of another protein (ORF33) was revised based on proteomic analyses [6]. This proteomic analysis also confirmed that the DNA polymerase (ORF17) has a putative frameshift or is a split variant of the DNA polymerase gene [5]. The revised genome arrangement based on these results is shown in Fig. 1C and has been updated in GenBank, along with the new protein designations, and appears under the original accession number: AY939843.

Our findings indicate that the ends of the P-SSP7 genome are similar in structure to those of T7. This suggests that the P-SSP7 phage is likely to be replicated, recombined into linear concatemers and packaged in a manner similar to that of T7 and other T7-like phages.

These results highlight the importance of the experimental determination of phage genome ends rather than relying on genome assembly programs. The latter automatically assemble genomes with rather long direct terminal repeats into circular molecules, as they do not take into consideration the possibility of the presence of terminal repeats even though they are often present in viral genomes. This is especially important to keep in mind in light of the high number of viral genomes from environmental isolates that are currently being sequenced.

## Materials and Methods

### Purification of High Molecular Weight Genomic DNA

P-SSP7 was propagated on *Prochlorococcus* MED4 grown on the seawater-based Pro99 medium [17] under a 14∶10 light:dark cycle at 10 µmol photon·m^−2^·s^−1^. A 1 L lysate, containing approximately 10^8^ phages·ml^−1^, was concentrated to 1 ml using Amicon Ultra 100 K centrifugal filters (Millipore) at 3000 Xg. The phage particles were embedded in a plug of low melting point agarose (SeaPlaque GTG, Lonza) and treated with 1 mg/ml proteinase K [18]. A slice of this agarose plug was run on 1% low melting point agarose (SeaPlaque GTG) in 1 X TAE and separated by pulse field gel electrophoresis (PFGE) using the CHEF-DR II PFGE machine (Bio-Rad). The running conditions were 6 V/cm with 5–15 s pulses, for 13 h at 14°C. The region containing the phage DNA (determined from the relative position to a Lambda marker) was cut from the gel without exposure to UV and extracted using GELase (Epicentre). Preparation of DNA using this method limits DNA shearing to a minimum and ensures that high molecular weight DNA is obtained.

### Cloning of P-SSP7 into a Fosmid Vector

High molecular weight genomic DNA was used to clone the P-SSP7 genome into the pCC1FOS fosmid using the CopyControl Fosmid Library Production kit (Epicentre Biotechnologies) following the manufacturer’s instructions. Briefly, the phage DNA was treated with T4 DNA polymerase and T4 polynucleotide kinase to generate blunt 5′ phosphorylated ends. The phage DNA was ligated into the Eco72 I site of the linearized and dephosphorylated fosmid. This site is flanked on both sides by BamHI restriction sites. The ligated DNA was packaged using the Lambda packaging extract and used to infect the EPI300-T1 *E. coli* strain.

To screen for clones that contained the entire P-SSP7 genome, PCR was performed using primer sets for the open reading frames at each end of the genome. The first primer set (ORF1-301F and ORF-588R) amplifies 307 bp from ORF1 at the left-hand end of the genome and the second primer set (ORF51-543F and ORF54-42R) amplifies 737 bp from ORFs 51 to 54 at the right-hand end of the genome. See Table 1 for all primer sequences. Fosmid DNA extracted from 120 *E. coli* transformants by standard alkaline lysis procedures [19] was used as template. One out of 120 fosmid clones, Fos5, contained both ORF1 and ORF51-54 as determined by PCR amplification and was used for sequencing (see below). The PCR reactions included 0.5 µM of each primer, 200 µM dNTPs, 1X OptiBuffer reaction buffer, 1 units of BIO-X-ACT Short DNA polymerase (Bioline), and 4 ng/µl of template DNA in a final reaction volume of 25 µl. PCR cycling conditions included an initial denaturation step for 5 minutes at 95°C followed by 40 cycles of denaturation for 30 seconds at 95°C, annealing for 30 seconds at 52°C, and elongation at 70°C for 1 minute and a final elongation step at 70°C for 5 minutes.

**Table 1:** PCR and sequencing primers used in this study.

Primer name	Primer sequence 5′-3′	Target	Reference
ORF1-301F	GATCTAGTGCAAGCTAACCA	P-SSP7	This study
ORF1-588R	GGCATACTACAACCTTACGT	P-SSP7	This study
ORF51-543F	CGACCCACAGTCAAGTGCTA	P-SSP7	This study
ORF54-42R	AGACCACATCTGGGTCTTCA	P-SSP7	This study
37R	CATCGTTAGGCATCGAGTA	P-SSP7	This study
44311R	TGTCAATTCGCCCAGATTCCAGGTA	P-SSP7	This study
43765F	ATCTCCATTTGTTGGTCGAT	P-SSP7	This study
T7	AATACGACTCACTATAG	Fosmid	This study
RP-pCC1	CTCGTATGTTGTGTGGAATTGTGAGC	Fosmid	This study
psbA_F	GTNGAYATHGAYGGNATHMGNGARCC	P-SSP7	[20]
psbA_R	GGRAARTTRTGNGCRTTNCKYTCRTGC	P-SSP7	Adapted from [20]
RepeatF	TATACGATGTACACAGGACGCA	P-SSP7	This study
RepeatR	CACAGGCGTGTCTGTATAAT	P-SSP7	This study

### Sequencing the Genome Ends

To determine the ends of the P-SSP7 genome, Sanger sequencing was carried out on P-SSP7 genomic DNA extracted directly from phage particles as well as on fosmid DNA containing the cloned P-SSP7 genome. For sequencing directly from the phage particle, high molecular weight genomic DNA extracted from the agarose plug was used as a template. Sequencing was carried out with the ORF1-588R and 37R primers (Table 1, Fig. 1). Genomic DNA was also digested by BamHI and PmeI restriction enzymes, and the 2 smallest fragments corresponding to the genome ends were extracted from the gel and used for sequencing with primers 44311R and 43765F (Table 1, Fig. 1). This served to separate the two repeat regions from the same large piece of DNA, and was necessary to overcome sequencing problems that arose using undigested genomic DNA with a primer positioned within ORF54. (Those reads were of low quality with many nucleotide positions remaining unresolved and inconsistent lengths received for different sequencing reactions.) Sequencing of the cloned P-SSP7 genome was carried out using primers T7 and RP-pCC1 (Table 1), positioned on the fosmid on either side of, and in the direction of the insert, as well as using a primer from within the phage genome (588R) to obtain sequence in the direction of the fosmid.

### Digestion and Southern Analyses

Restriction enzyme and Southern analyses were carried out on both P-SSP7 genomic DNA extracted from phage particles and the cloned P-SSP7 genome. The DNA (0.5 µg per reaction) was digested with BamHI and with a combination of BamHI and PmeI (New England Biolabs). The digested DNA was run on 1% Seakem Gold agarose (Lonza) in 0.5X TBE and separated by pulse field gel electrophoresis using the running conditions described above. Fragment size markers were the 1 kb DNA ladder and phage Lambda DNA cut with HindIII. For Southern analyses, the digested DNA was transferred onto NytranN nylon membranes (Schleicher & Schuell BioScience) by capillary alkaline transfer using 0.4 N NaOH as the transfer solution.

The DNA fragments used for probe preparation were amplified by PCR. The ORF1 probe was amplified with the ORF1-301F and ORF1-588R primers; the ORF51-ORF54 probe was amplified with the ORF51-543F and ORF54-42R primers; the *psbA* probe was amplified with the psbA_F and psbA_R primers; and the repeat region was amplified using the RepeatF and RepeatR primers. See Table 1 for all primer sequences. The PCR conditions were as described above except that the annealing temperature was 55°C. PCR products were excised from 2% agarose gels and purified with a MinElute gel extraction kit (Qiagen). The probes were directly labeled with the alkaline phosphatase enzyme and detected on the membrane after decomposition of the dioxetane chemiluminescent substrate using Amersham’s AlkPhos Direct Labeling and Detection Systems kit with CDP-Star (GE Healthcare) and exposure to a BioMax MS photographic film (Kodak).
